# An Analysis of the Vulnerability of Two Common Deep Learning-Based Medical Image Segmentation Techniques to Model Inversion Attacks

**DOI:** 10.3390/s21113874

**Published:** 2021-06-04

**Authors:** Nagesh Subbanna, Matthias Wilms, Anup Tuladhar, Nils D. Forkert

**Affiliations:** 1 Department of Radiology, University of Calgary, Calgary, AB T2N 1N4, Canada; matthias.wilms@ucalgary.ca (M.W.); anup.tuladhar@ucalgary.ca (A.T.); nils.forkert@ucalgary.ca (N.D.F.); 2 Hotchkiss Brain Institute, University of Calgary, Calgary, AB T2N 1N4, Canada; 3 Alberta Children’s Hospital Research Institute, University of Calgary, Calgary, AB T2N 1N4, Canada

**Keywords:** medical imaging, deep neural networks, inversion attacks, patient privacy

## Abstract

Recent research in computer vision has shown that original images used for training of deep learning models can be reconstructed using so-called inversion attacks. However, the feasibility of this attack type has not been investigated for complex 3D medical images. Thus, the aim of this study was to examine the vulnerability of deep learning techniques used in medical imaging to model inversion attacks and investigate multiple quantitative metrics to evaluate the quality of the reconstructed images. For the development and evaluation of model inversion attacks, the public LPBA40 database consisting of 40 brain MRI scans with corresponding segmentations of the gyri and deep grey matter brain structures were used to train two popular deep convolutional neural networks, namely a U-Net and SegNet, and corresponding inversion decoders. Matthews correlation coefficient, the structural similarity index measure (SSIM), and the magnitude of the deformation field resulting from non-linear registration of the original and reconstructed images were used to evaluate the reconstruction accuracy. A comparison of the similarity metrics revealed that the SSIM is best suited to evaluate the reconstruction accuray, followed closely by the magnitude of the deformation field. The quantitative evaluation of the reconstructed images revealed SSIM scores of 0.73±0.12 and 0.61±0.12 for the U-Net and the SegNet, respectively. The qualitative evaluation showed that training images can be reconstructed with some degradation due to blurring but can be correctly matched to the original images in the majority of the cases. In conclusion, the results of this study indicate that it is possible to reconstruct patient data used for training of convolutional neural networks and that the SSIM is a good metric to assess the reconstruction accuracy.

## 1. Introduction

The number of deep learning techniques employed to solve segmentation and classification tasks in medical imaging is increasing by the day [1,2]. Deep learning techniques have revolutionized medical image analysis as they can learn from large amounts of data and often offer advantages in accuracy compared to conventional machine learning methods. Consequently, many commercial systems using deep learning models are being deployed in practice for tasks, such as segmentation and classification [3], while many other non-commercial models are made publicly available. Overall, deep learning is expected to play a leading role in the future of precision medicine [4].

One of the most important aspects when using medical images to train deep neural networks is patient data confidentiality. Within this context, patient data does not only include the imaging data itself (X-ray, CT, MRI, PET, etc.), but may also include other relevant attributes used for training of deep neural networks, such as age, sex, medical condition (high blood pressure, diabetes, etc.), and life style factors (smoking, alcohol, etc.). Within this context, even from ‘a blurry version of the original scan’, patient-identifying information may be inferred based on, for example, specific pathologies (e.g., a rare tumor) or using advanced facial recognition methods [5]. This is especially a severe risk as previous research has also shown that it might be possible to identify the imaging center from images alone, which allows to locally restrict persons of interest. More precisely, it is well known in the medical imaging community that specific scanners and/or imaging protocols allow to identify a data acquisition site based on image-only information [6]. The combination of this with clinical data used for training that might be also recoverable could reveal a significant amount of sensitive data about a single patient. Thus, maintaining the confidentiality of this patient data (images, attributes, etc.) at every stage of information processing is paramount.

Until recently, patient data confidentiality was thought to be protected in trained deep learning models, which were assumed to be black boxes and learn from a plethora of information. Recent research in computer vision [7,8] has shown that it is possible to reconstruct, for example, facial photos used for training of a deep neural network to the extent that allows a person to be identified. However, it remains unknown if such methods could also be used to reconstruct complex 3D medical imaging data.

The primary aim of this work is to examine the vulnerability of two deep learning segmentation networks commonly used in medical imaging to model inversion attacks. The two network architectures chosen for this purpose are the SegNet [9] and the U-Net [10]. These two architectures were selected as they are extremely popular, as evidenced by the thousands of citations each technique has received. Additionally, these two models can be both classified as encoder-decoder techniques that use a latent space representation from which the original volumes can be reconstructed. The latent space provides the most compressed representation of the images in the network.

The main contributions of this work are two-fold:

- First, we investigated the feasibility of model inversion attacks on two well established segmentation models, namely a U-net and a SegNet, trained on 3D medical images.
- Second, we implemented and compared multiple similarity metrics, which can be used to evaluate the quality of the reconstructed medical images. This is especially important for future research endeavors in this domain as metrics in the computer vision domain might not be applicable or might not be ideal when applied to 3D grayscale images from the medical domain.

This work is based on our recent conference paper [11] and extends it in four ways:

1. Addition of the SegNet architecture.
2. Use of a larger database of 3D brain MR images with manually defined labels for multiple brain structures (LPBA40 [12]).
3. Segmentation of brain structures as the task being analyzed instead of ischaemic stroke lesion segmentation.
4. Addition of multiple evaluation metrics to measure the reconstruction quality and possibility to identify single subjects based on the reconstructed images.

## 2. Literature Review

In general, there are multiple types of deep learning model attacks that attempt to reconstruct the original data used for the training of deep neural networks. The first type is the membership inference type attack. This type of attack [13,14] aims to determine if a particular image is part of the training set. Another type of attack is the attribute inference attack [15]. In this type of attack, some of the attributes are available through public databases, or by other means, and the attacker tries to obtain the sensitive attributes of the person [16,17]. A third type of attack is the model stealing attack [18,19] with the primary aim of inferring the parameters and hyper-parameters of the deep learning model. The last attack type is the model inversion attack [7,8,20]. In this case, an attacker tries to reconstruct the original data used for training of the deep learning model. Given the vast number of trained deep learning models that are publicly available, the model inversion attack is potentially the most relevant regarding patient privacy issues in medical applications.

Generally, model inversion attacks take advantage of two important properties of deep learning techniques. First, many deep learning techniques learn an abstract representation of the images, which are then processed across multiple stages into the output (e.g., classification or segmentation). Second, compared to the large number of parameters that are optimized during model training, the models are typically trained on rather small and often imbalanced training sets.

This second problem is more severe in medical imaging compared to the computer vision problems mentioned above since the training is typically performed using small medical imaging datasets on the order of a few tens or hundreds of images, which increases the risk of overfitting. Both of these properties are utilized during an inversion attack of the deep learning models. Based on the amount of information available to the attacker, attacks are categorized into white box attacks and black box attacks. In white box attacks, the deep learning network parameters are known to the attacker, which is not the case in black box attacks. Given the type and information available of many publicly available deep learning models in medical imaging, the more simple white box attacks pose a significant threat to patient privacy.

While there has been some research on the reconstruction of images used for training of deep learning models in computer vision, the number of studies that focus on model inversion attacks for medical images is rather limited.

## 3. Materials and Methods

### 3.1. Segmentation Models

**Figure 1:**
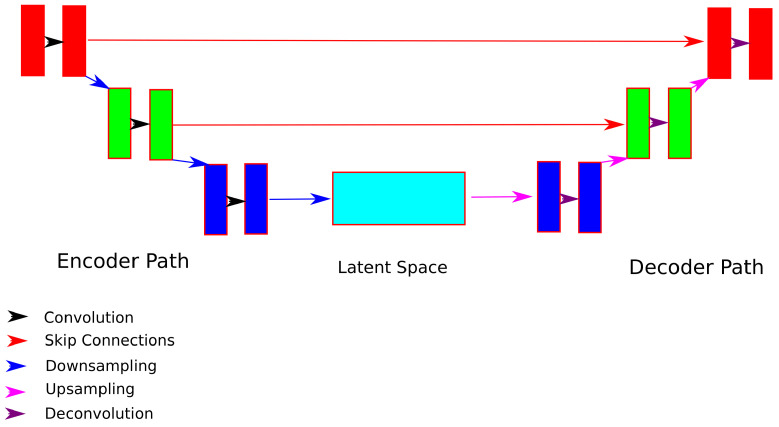
The schematic diagram of a U-Net architecture. The model is divided into a contraction path and an expansion path, with the latent space representation generated at the end of the contraction path. The contraction is comprised of a convolution with a set of filters, followed by a downsampling operation that reduces the size of the image. This process is continued until the latent space is reached. In the expanding path, there is an upsampling followed by a deconvolution. This process is continued until the desired output image is reached. The corresponding layers of the expansion and contraction paths are connected using so-called skip connections.

The U-Net model is a popular convolutional neural network. The architecture consists of a contracting path to capture the context in the images and a symmetric expanding path to enable precise localization. The schematic illustration of a U-Net is shown in Figure 1. In the U-Net, the contracting path works based on convolution kernels of size x×x×x, each followed by a non-linear activation function (ReLU here), and a max-pooling operation over a y×y×y block to downsample the image. The number of filters *z* doubles at every contraction, and there are a total of *n* layers. In the expanding path, there is a corresponding up-convolution at every step. A concatenation of the correspondingly cropped feature maps from the contracting path is achieved using the skip connections between the corresponding levels. Finally, there are again convolutions with x×x×x kernels, each followed by a ReLU activation.

**Figure 2:**
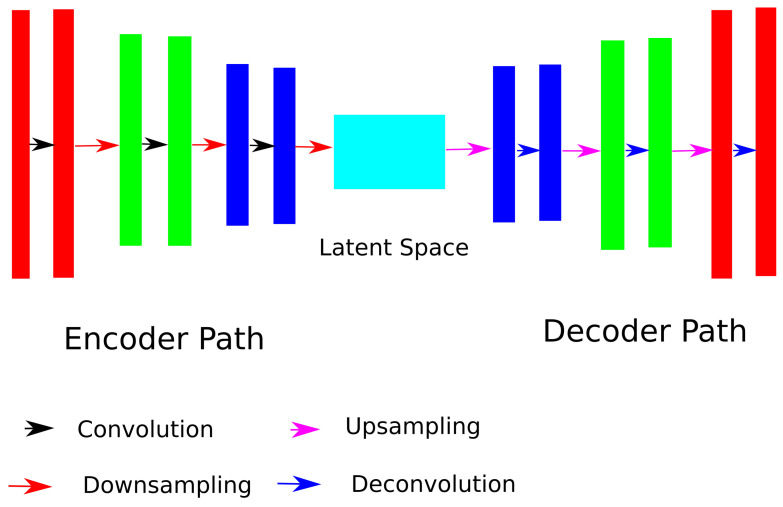
The schematic diagram of the SegNet architecture. This model is also divided into the expansion and contraction paths but does not include any skip connections.

The SegNet architecture also follows an encoder-decoder network architecture principle (shown in Figure 2), with the encoder path consisting of the earlier layers of the VGG16 network [21]. In comparison to the U-Net architecture, the fully convolutional approach is dropped in favor of higher resolution maps at the deepest encoder output. Each encoder performs a convolution over a x×x×x window with a filter bank, followed by a non-linear activation function (ReLU here), and a max pooling operation over a y×y×y window. Similar to the U-Net, the SegNet architecture starts with *z* filters that double at each contraction step with a total of *n* steps. Each encoder has a corresponding decoder in the decoder path, which performs the inverse operation. The final decoder output is typically fed to a multiclass soft-max classifier to produce class probabilities for each pixel independently.

### 3.2. Inversion Attack

The inversion attack scenario used in this work was first described for computer vision applications [7,8] and is based on the following assumptions that hold true for most deep learning models that are publicly available and follow an encoder-decoder architecture:

1. The attacker can access the latent space representation of arbitrary input images.
2. The attacker knows the encoder’s architecture that is used to generate the latent space information of the images.
3. The attacker can train a separate decoder to reconstruct images based on the latent space representations.

**Figure 3:**
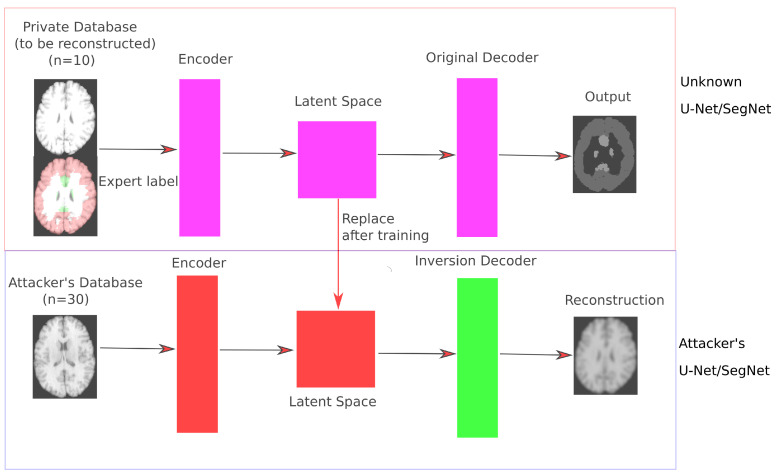
Schematic flowchart of an inversion attack. The original network includes the encoder, which leads to the latent space, and the corresponding decoder path that produces the task-specific output. Using the latent space information and an independent database of training images, a separate (inversion) decoder is trained to reconstruct the original images. This decoder is trained to minimize the difference between the original image and the reconstructed image.

Briefly described, model inversion attack techniques train a separate inversion decoder that is able to reconstruct images based on their latent space representation (Figure 3). As the attacker has no access to the original training images, training of the inversion decoder is based on an independent database of images $X_{attack}$. Once the inversion decoder is trained, the attacker tries to reconstruct images from the original private training database. The detailed setup used in this study is described in the following.

For this study, we assume a trained encoder-decoder segmentation network to be given. For a 3D input image volume X(x,y,z), the trained encoder Enc(X) maps the image to its latent space representation Z=Enc(X). The decoder part of the network then maps this latent space information *Z* to the output space *Y*, in this case, a segmentation image, using the task-specific decoder Y=Dec(Z). This can be written as X→Enc(X)→Z→Dec(Z)→Y.

Model inversion now aims to create a separate inversion decoder that is optimized by minimizing the difference between an original image and its reconstruction from the latent space representation as shown in Figure 3. More formally, given the attacker’s database $X_{attack}$, the goal is to reconstruct each image $\left\{X\in X_{attack}\right\}$ based on the latent space information *Z* obtained by the encoder path Z=Enc(X). This process can be seen as a way of reconstructing the original image from its most compressed version. Training the inversion decoder $\hat{X}=\hat{D}\left(Z\right)$ focuses on learning network parameters that minimize the squared norm between the original image *X* and its reconstructed version $\hat{X}$:

(1) $$L=Enc_{X\in X_{attack},Z}||\hat{X}-X|{|}^{2}.$$

This newly trained inversion decoder essentially inverts the encoder of the segmentation network and is utilized by the attacker to reconstruct images from the original training database $X_{private}$ to which the attacker has no access. Successful reconstruction would compromise the data privacy of patients included in $X_{private}$ as it may be possible to identify them from the reconstructions.

To simplify our analysis and to be able to focus on the reconstruction aspect of a model inversion attack, we assume the latent space representations of all images from the original training database $X_{private}$ to be given for the attack. In a real-world scenario, the attacker would have to obtain them in an additional step. This could be achieved via a systematic analysis of the segmentation networks’ latent space and how the task-specific decoder converts latent representations into segmentations. Particularly, in scenarios where $X_{private}$ is small, it can be assumed that the segmentation network is more confident about segmentations for latent space elements that are close to the real training data. This information can then be used to at least approximately infer latent space representations of the real training images to perform the attack.

### 3.3. Implementation Details

**Figure 4:**
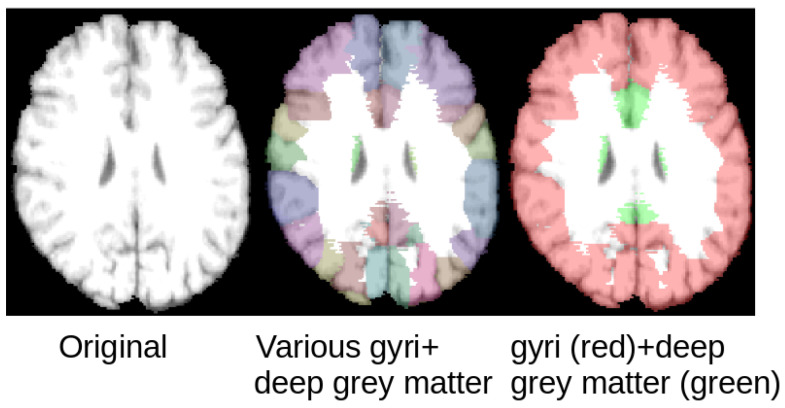
Selected slice from a MRI dataset from the LPBA40 database (**left**) with expert labels superimposed on the original slice (**middle**) and the fused labels used in this work (**right**).

The inversion attack scenario described above was developed and evaluated using the LPBA40 database, which is comprised of 40 brain MRI datasets [12]. All images consist of 181×217×181 voxels of isotropic size (1 mm × 1 mm × 1 mm in the x, y, and z directions). All datasets were transformed to a common space using rigid registration by the data providers. For simplicity reasons, this study uses three tissue classes for the segmentation problem, with the three classes being gyri, deep grey matter, and the remaining brain tissue (see Figure 4). Therefore, the expert segmentation labels of various gyri and deep grey matter structures available as part of the LPBA40 database were used to generate the ground truth segmentations required for model training of the SegNet and U-Net model. More precisely, the various gyri labels were combined to a single gyri class, the various deep grey matter structures to one deep grey matter class, and all remaining brain tissue to the third tissue class. Thus, the SegNet and U-Net models were trained with the goal of segmenting those three tissue classes.

For preprocessing, all 3D images of size 181×217×181 were padded to 224×224×224 voxels to ensure that the number of voxels are not fractions after the processing given the four encoder and decoder layers. Thus, the number of voxels in each dimension are required to be divisible by 16. After this, all images were processed using a N3 bias field correction [22] and normalized using the method suggested in Reference [23].

The encoder path comprises of 3×3×3 convolution windows followed by rectified linear unit (ReLU) activation functions. The output of the ReLU is then passed on to a 2×2×2 max pooling window. This general structure is repeated in each layer. A total of 4 encoding and decoding layers are used in the networks. The number of filters begins at 16 and doubles at each layer. The decoder path comprises of an upsampling to double the size of the image and a deconvolution to the next layer. In case of the U-Net only, skip connections are added between corresponding layers. Finally, the output of the decoder is a segmentation image of the same dimensions as the original image.

### 3.4. Experiments and Evaluation Metrics

Four-fold cross validation was used to train the original networks and corresponding inversion decoders. More precisely, in each iteration, 10 images were used as the private database for training the segmentation CNN models (U-Net and SegNet). The remaining 30 images were used to train the inversion decoder aiming to reconstruct the 10 images originally used for training, which were compared qualitatively and quantitatively to the private original images used for training.

In our experiments, we used the full brain MRI scans, as well as skull-stripped versions. Proportionally, the volume of the brain segmented in the non-skull-stripped images is smaller than the volume of the image segmented in skull-stripped images. Thus, this experiment enables an analysis if the change in proportion of the image segmented has an effect on the reconstruction quality.

Four separate evaluation metrics were implemented in this work to measure the reconstruction success in our inversion attack scenario: The first metric is the frequently used Matthews correlation coefficient (MCC) [8] between the original and the reconstructed image. The second one is the structural similarity coefficient (SSIM) [24], which is defined as a function of luminance, contrast, and structure of the two images being compared, and ranges between 0 and 1, where 0 denotes no similarity, and 1 denotes that the two images are identical. In addition to the two intensity-based similarity measures, we also evaluated the use of non-linear deformation fields computed using ANTs [25] to measure reconstruction success with respect to the different shapes in the images. Here, we compute the mean norm of all displacement vectors resulting from the registration of the reconstructed image to the images in the training dataset. In this case, we expect that the smallest mean norm of all displacement vectors will be found when comparing an reconstructed image to the correct original image. Finally, we also segmented the reconstructed images using the same U-Net and SegNet and computed the Dice similarity scores to investigate if the same images can be re-used for segmentation, and to what extent they resemble the segmentation of the original images. This can be seen as a way of combining intensity- and shape-based information when assessing reconstruction quality.

## 4. Results

### 4.1. Qualitative Results

**Figure 5:**
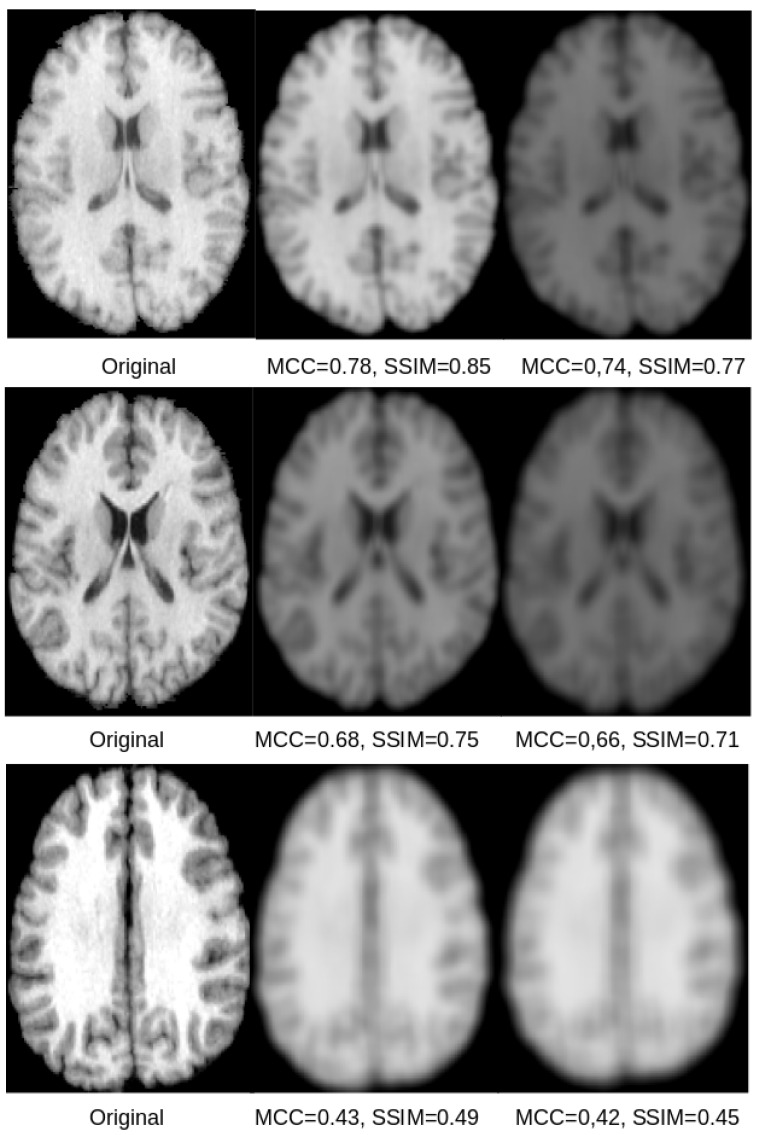
The original skull-stripped image (**left**) and the corresponding reconstruction from the latent space of the U-Net (**center**) and SegNet (**right**) for three different subjects. For the subject shown in the top row, the reconstructions are sharp and there is only a scaling difference between the original and the reconstructed images. In case of the SegNet reconstruction, some minor blurring can be seen. For the subject shown in the middle row, the reconstructions show some significant degradation due to blurring, but they are still clear and recognizable. For the subject shown in the bottom row, the reconstructions show severe blurring. The corresponding SSIM and MCC scores reflect those qualitative impressions, whereas the SSIM seems generally better suited and more sensitive.

**Figure 6:**
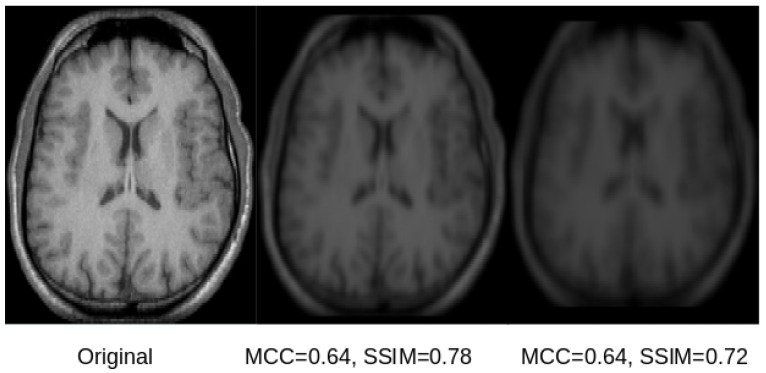
A selected slice from an original MRI dataset without skull stripping (**left**) and its reconstruction from the latent space of the U-Net (**center**) and SegNet (**right**). As can be observed in the image, the original and the reconstructed images are overall similar. However, the reconstruction from the SegNet model is more blurry, which is reflected by lower a SSIM score, while the MCC is very similar for both reconstructions, not reflecting the differences in reconstruction quality.

Exemplary qualitative results for U-Net and SegNet-based reconstructions for the skull-stripped and original images are shown in Figure 5 and Figure 6, respectively. Those results indicate that both SSIM and MCC in general reflect the visual quality of the reconstructed image in comparison to the original image. Both measures assign higher values to visually better reconstructions that are less blurry. However, the SSIM metric appears better suited and more sensitive to image blurring, making it the better metric for this purpose.

### 4.2. Quantitative Results

#### 4.2.1. Mean Cross Correlation and Structural Similarity

MCC- and SSIM-based quantitative results of all experiments are summarized in Table 1. For the U-Net, the mean MCC and SSIM are 0.60±0.14 and 0.73±0.12, respectively, for skull-stripped images. For the SegNet, the MCC and the SSIM are 0.53±0.13 and 0.61±0.12, respectively. For non-skull-stripped images, the mean MCC and SSIM for the U-Net are 0.43±0.15 and 0.50±0.11, respectively. The corresponding mean MCC and the SSIM for the SegNet are 0.39±13 and 0.43±0.13, respectively. Thus, the results suggest that images can be better reconstructed from a trained U-net compared to a trained SegNet and with better results for skull-stripped images compared to the original images without skull stripping.

So far, we have shown that SSIM and MCC values are both useful indicators of the visual quality of the reconstruction. However, in order to really determine reconstruction success based on these measures, it is necessary to establish baseline values and investigate if SSIM and MCC values comparing a reconstructed image and its original are higher than when comparing the reconstructed image to images of other subjects. Therefore, we performed an additional experiment in which we computed the SSIM and MCC values of each reconstructed (skull-stripped) image with all other images in the test dataset (see Table 2). As can be seen in Table 2, the SSIM comparing a reconstructed image with its true corresponding original image is on average 0.23 better for the U-Net in comparison to the mean SSIM comparing a reconstructed image to the original images from all other subjects. This general finding is also true for the SegNet reconstructions with an improvement of 0.12.

Next, we ranked the different SSIM and MCC results to investigate if the similarity measurements comparing a reconstructed (skull-stripped) image with its true original image are always higher than the values achieved for other images. The corresponding results for this experiment are summarized in Table 3 and indicate that the SSIM metric is able to reliably identify the correct original image in the dataset in most cases. More precisely, the SSIM of the reconstructed image was highest in 30 of the 40 cases for the corresponding original image, second highest in 5 cases, third highest in 2 cases, fourth highest in 2 cases, and fifth highest in 1 case for the U-net, leading to an average rank of 1.5. In case of the SegNet reconstructions, the SSIM compared to the true original image was the highest in 26 of the 40 cases, second highest in 8 cases, third highest in 3 cases, fourth highest in 2 cases, and sixth highest in 1 case, with an average rank of 1.6. The results of this experiment show that it is a realistic risk that it is possible to reconstruct patient data used for training of the CNN that allows identifying patients with an increased risk in U-Net architectures.

#### 4.2.2. Deformation Field

**Figure 7:**
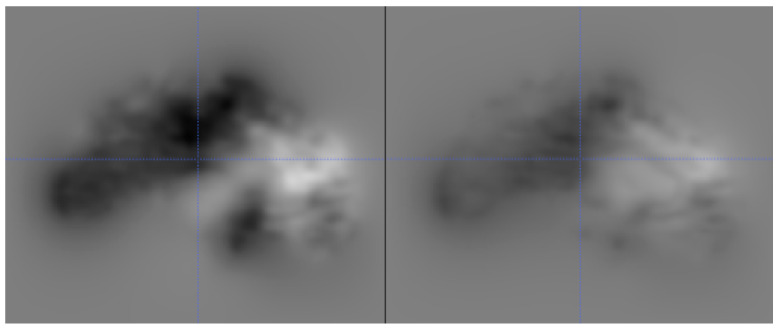
An example of a good reconstruction (**left**), which has much smaller, and more localized deformations compared to an example of a bad reconstruction (**right**), which requires large deformations everywhere. Smaller deformations are displayed by the darker intensities, while the larger deformations are displayed by lighter intensities.

Apart from the two main intensity-based similarity metrics described above, we also computed the norm of all displacement vectors of the deformation field after registration of the original image and the corresponding reconstruction to assess the success of the inversion attack. Therefore, we affinely registered each reconstructed image to the true original image and all other original images of the database using ANTs [25] and mutual information as the similarity measure. In the second phase, each affine registration was further refined using a non-linear registration optimized using the cross-correlation similarity measure implemented in ANTs. In this case, it is assumed that the norm of all displacement vectors of the deformation field should be smallest for the true original image of a reconstruction when compared to the corresponding values for all other images. Exemplary deformations are shown in Figure 7. Using this approach, the mean rank (for skull-stripped images) of the true original image is 1.8 for the U-net, as well as SegNet. More precisely, 24 reconstructed datasets could be accurately matched to the true original image for the U-Net, while 22 datasets could be matched correctly for the SegNet reconstructions.

#### 4.2.3. Reconstruction Dice Similarity Coefficient

For the final experiment, the reconstructed images (skull stripped) were segmented using the originally trained U-net and SegNet to determine how the segmentations compare against those generated based on the true original images. In both cases, the Dice similarity metric was computed by comparison of the CNN segmentation to the manual ground truth segmentation. This experimental setup led to a mean Dice value of 0.53±0.14 for the U-Net when the original images are used and 0.37±0.17 for the reconstructed data sets. For the SegNet, an average Dice score of 0.48±0.13 was achieved for the original images and 0.32±0.12 for the reconstructed images. The computed Dice scores indicate that there is a significant degradation of the segmentation when the reconstructed images are utilized, suggesting that the Dice score is not a good metric to evaluate reconstruction accuracy.

## 5. Discussion

The aims of this paper were twofold: (1) We wanted to examine if popular segmentation CNN architectures in medical imaging (U-Net and SegNet) can be attacked via model inversion attacks. (2) We aimed at examining the ability of different similarity measures to quantitatively assess the success of the attack by comparing the reconstructed image with its original.

Based on the visual results exemplified in Figure 5 and Figure 6, it can be concluded that all reconstructions show some degree of blurriness. The overall reconstruction quality including the blurriness is best reflected in the SSIM scores, which range between 0.39 and 0.88. Overall, the results of this study show that reconstruction of the original images used for training of CNN models is possible, but is accompanied by some amount of blurring. This finding is also in line with the results from model inversion attack studies in computer vision [8]. The results of this study also suggest that the reconstructions from the U-Net are better than those for the SegNet. A potential reason for this finding could be the skip-connections of the U-Net.

The results of this study also suggest that the proportion of the volume that is actually segmented by the deep learning technique is relevant with respect to the reconstruction quality. More precisely, in case of the skull-stripped images, where the proportion of relevant image voxels that have been segmented is larger, the reconstruction is considerably better, which holds true for the U-Net and for the SegNet. This shows that CNN models trained to segment larger regions of medical images are easier to attack than CNN models trained to segment small regions, such as brain lesions.

The most commonly used metric in the computer vision [7,8] and in medical imaging [15] domain for image similarity assessment of reconstructions is the MCC, which examines the correlation between the grey levels of the original and the reconstructed images. However, it was found in this study that many images are reconstructed with non-linear intensity differences, which is not well captured by a simple correlation metric. Furthermore, blurring is also not as well reflected in the MCC as in the SSIM, which not only uses intensity, but also contrast and structure, thus better capturing the visual similarities. Consequently, the MCC is not a good metric to quantitatively assess the visual similarity of the reconstructed image for this specific problem. In Reference [26], the authors discuss the various weaknesses of correlation coefficients, which hold true for the results in this study. Furthermore, trying to match the reconstructed image with the corresponding true original using the MCC score results in the correct match of only 16 and 14 of the 40 images for the U-Net and the SegNet, respectively. The SSIM-based matching performed considerably better and resulted in correct matches in 30 and 26 cases, respectively. The finding that the SSIM is the best overall indicator of the reconstruction quality is also consistent with the visual examination. The better the reconstruction looks visually, the higher the SSIM seems to be. The deformation field measure is generally also a suitable metric for this purpose, giving good matches, but there are cases where large local deformations heavily influence the overall score. Additionally, this similarity metric is rather computationally expensive to compute due to the non-linear registration required. The final metric investigated, the Dice similarity coefficient of the reconstructed images, cannot be used to directly match a reconstructed image with the corresponding true original image. However, it can be a useful score to obtain an impression of the overall goodness of the reconstruction.

One important limitation of this study is that the training and the test sets were part of the same overall database. Thus, it remains to be seen whether the same levels of consistency of reconstruction can be obtained with databases obtained under different acquisition conditions. To model this potential problem in our experimental setup, we also trained a separate encoder when training the network used for inversion attack in this work and then replaced the latent space. Another important limitation is that the CNN models were only used based on brain MRI datasets of healthy subjects. However, we believe that our study is an important first step in analyzing privacy problems related to deep learning models in medical image analysis in general and that it shows that training data leakage is a serious problem. Furthermore, we are confident that the results of this study also hold true for other imaging modalities or body parts. This should be verified in future studies where it would also be interesting to add clinical metadata (e.g., patient age, sex, comorbidities) to the analysis. Being able to not only reconstruct imaging data, but also clinical metadata, would pose an even more severe privacy breach.

## 6. Conclusions

The results of this paper show that it is possible to reconstruct a brain MRI from a trained U-Net and SegNet, albeit with some degradation due to blurring, provided that the U-Net and the SegNet have been trained to segment large parts of the original volume. Despite the blurring, the reconstructed images can be correctly matched to the original images in the majority of the cases. Finally, examining the various scores for measuring the quality of the reconstruction suggests that the SSIM score reflects the reconstruction success best and should be used in future studies in this domain.

## Figures and Tables

**Table 1:** The results of the different experiments that have been performed for the MCC and SSIM scores comparing the original images with the corresponding reconstructed images.

Metric	U-Net-Skull Stripped	SegNet-Skull Stripped	U-Net with Skull	SegNet with Skull
MCC	0.60±0.14	0.53±0.13	0.43±0.15	0.39±0.13
SSIM	0.73±0.12	0.61±0.12	0.50±0.11	0.43±0.13

**Table 2:** Comparison between the mean SSIM and MCC scores of the reconstructed images with the true original images and corresponding mean scores of the reconstructed images with the images from all other patients in the database. Skull-stripped images were used for these experiments.

Metric	SSIM with Originals	SSIM with Others	MCC with Originals	MCC with Others
U-Net	0.73±0.12	0.50±0.23	0.60±0.14	0.52±0.22
SegNet	0.61±0.12	0.49±0.23	0.52±0.13	0.45±0.20

**Table 3:** Number of correct matches of reconstructed images to the corresponding true original images for n = 40 datasets.

Experiment	U-Net (Skull Stripped)	SegNet (Skull Stripped)
MCC match	16	14
SSIM match	30	26
Deformation field match	24	22

## Data Availability

The LPBA40 database [12] is publicly available. The code for the deep learning inversion attacks will be made available after acceptance of this paper.
